# Two sub-cultures of explanatory computational psychiatry

**DOI:** 10.1038/s41380-024-02639-w

**Published:** 2024-07-02

**Authors:** Adrian Kind, Peter Dayan

**Affiliations:** 1 Research Training Group 2386, Berlin School of Mind and Brain, Humboldt-Universität Zu Berlin, Berlin, Germany; 2 Department of Philosophy, Otto- von-Guericke University Magdeburg, Magdeburg, Germany; 3 Research Division of Mind and Brain, Department of Psychiatry and Psychotherapy Campus Charité Mitte, Charité – Universitätsmedizin Berlin, Berlin, Germany; 4 Max Planck Institute for Biological Cybernetics, Tübingen, Germany; 5 University of Tübingen, Tübingen, Germany

**Keywords:** Neuroscience, Diagnostic markers

## Introduction

In attempting to explain the properties and behavior of an evolved artefact with the breathtaking complexity of the human brain, dissociating conventional causes and effects is like pulling apart an autosarcophagic snake. To paint a representative picture, we need a palette of explanatory languages capable of portraying the multifarious interactions within a complex system at suitable levels of abstraction. In particular, in addition to conventional causal explanations, the philosophy of science offers a framework of so-called constitutive explanations. Here, we motivate, describe, differentiate and discuss the use of causal and constitutive explanations in the field of computational psychiatry.

As a starting point, we take Bennett et al.´s [1]. influential paper entitled “*Two Cultures of Computational Psychiatry*”, which structures theoretical efforts in computational psychiatry. These authors distinguish two broad approaches in the field: predictive modeling and explanatory modeling. Predictive modeling “aims to predict what outputs a data-generating process will produce from a given set of inputs while treating the process itself as a black box” [1](p.1). This approach is mainly used to predict clinical outcomes, but without attempting to understand how these outcomes are generated. By contrast, explanatory modeling in the context of computational psychiatry “uses the pattern of outputs and inputs to explain how the data-generating process works. In [computational] psychiatry, the data-generating processes are the psychological and neurobiological mechanisms whose maladaptive operations produce psychiatric dysfunction” [1](p.1). This approach “focuses on statistical models (expressed as equations) that define interacting processes with parameters that putatively correspond to neural computations” [1](p.1). To introduce and discuss the distinction between causal and constitutive explanations, we propose that they define two sub-cultures as a further refinement of explanatory modeling.

## Causal and constitutive models

Consider a person who is so risk averse that, through experiencing outcomes, they come to avoid taking what is a 50/50 chance of winning $10 or losing $2. One causal explanation of this behavior is that the person has learned an average subjective utility for this chance that is negative, rather than the objective mean of $4. This cause could be reflected in below-baseline activity in some value-representing region of the brain. However, as an explanation this is thin gruel, not the least since it does not mandate predictions about risk avoidance in other circumstances. Analogously: saying that pulling the trigger caused a gun to fire may be a true causal explanation, it however does not help us to understand how the gun is constructed in such a way that pulling its trigger makes it fire. Following this intuition, three (of various) alternative accounts to explain the person’s behavior that are abroad in computational psychiatry are that: (i) each loss of $2 looms larger than each gain of $10 [2]; or (ii) that the person learns more from negative than positive outcomes in general [3]; or (iii) that they stick too rigidly to a prior distribution that favors bad over good outcomes (the flip side of Stankevicius et al) [4]. We argue that these three accounts are best seen as offering a different kind of so-called constitutive explanation.

Let us point out the gist of the difference between causal and a constitutive explanation based on our example. A causal explanation employs a chain of causally linked events as explanans (the occurrences leading to learn a certain average subjective utility and the corresponding neural events mirroring this process), to explain a state or occurrence chosen as explanandum (the decision not to take a 50/50 chance of winning $10 or losing $2) [5]. A constitutive explanation on the other hand addresses its explanandum by “abstract[ing] away from the behavior and orchestrated activities of [a system’s] parts,” and instead asking “how the system has a capacity for this kind of behavior” [6](p.122). This way these parts and their setup plays the role of explanans. We can see the system as having the disposition to operate in a particular way (coming to reject gambles that most people would come to accept) because of aspects of its parts (representing utilities, or learning rules, or priors) and their organization having its own realization in neural activity or rules for changing synaptic weights [7].

Of course, it is particularly the differences between these accounts that are most informative. The difference lies in how the causal capacity of the system (making a certain choice) is explained. A causal explanation spells out a stepwise causal process detailing at various levels of explanation how one (neural) event causes another, and another, until the output (choice) is produced. This provides us with a causal aetiology of the behavior of interest – an account of why the event that is intended to be explained actually occurred on a particular occasion. On the other hand, a constitutive explanation indicates how the causally relevant components of the system and their organization instantiate the causal capacity of making this behavior happen – an account of the features of the system that generated the event make the system have the disposition to act that way given some triggering conditions. In other words, causal explanations give us a ‘why-story’ which presents a causal chain of events leading to the outcome of interest, while a constitutive explanation, rather than presenting us with a chain of events, tells us how the system, in virtue of having certain properties, has the disposition to carry out a specific action.

It is important to note that while constitutive and causal explanations, as outlined above, are different types of explanations for causal events in a system, they are not epistemologically independent. We need some insight into the causal processes of a system to provide an account of its dispositions. However, the scope of the required insights will generally be more modest at this more abstract level. Thus, constitutive explanations are a lower hanging fruit in terms of the insight required into a system’s causal makeup. Distinguishing the explanations appropriately makes clearer how much farther we have to go to generate fully causal explanations.

Both types of explanatory models enjoy two additional dimensions of analysis. One dimension is the conventional notion of reduction in natural sciences, with phenomena described more abstractly at one level resulting from mechanisms operating at a finer level (typically of time and/or space). For constitutive models associated with over-weighting of negative over positive outcomes, we could imagine, for instance, the interactions between lower level systems with adaptive set points in which excitation and inhibition are unbalanced. However, the same disposition could come from other lower level (dis)organization [8].

The other dimension of analysis concerns the theoretical conviction that brains are solving what can be seen as computational and control theoretic problems associated with maintaining physical and social homeostasis over the long run. It is this perspective that provides semantics to the representational interpretations of neural activity and the neural transformations governing those representations. Thus, the excitation and inhibition mentioned above might represent the valance of an actual monetary outcome and the valence of a prediction of this outcome respectively, with the imbalance leading to a form of quantile or expectile prediction that embodies risk sensitivity [3]. It can be helpful to think about these computational descriptions as providing a form of interpretative patina around the conventional reduction. In fact, our descriptions of the causal and constitutive models has already, as is near ubiquitous for explanatory computational psychiatry, been described in these terms [9]. As the computational description can be thought of as an interpretative patina that can provide us with constitutive as well as causal explanations, one may point out that the option for providing such explanations is not limited to the computational level but exists across levels of explanation. If we think of Marr’s level ontology, this means that causal as well as constitutive explanations can also be provided at the algorithmic and implementational levels.

The combination of these two additional dimensions: reduction in terms of more fine-grained mechanisms, and the representational interpretation of the contributions of this mechanism in terms of computational description, is particularly important when trying to link radically different facets in psychiatry – for instance pharmacotherapy and cognitive behavioral or psychotherapies. This point has recently been emphasized in philosophy of neuroscience by pointing out that to succeed in providing inter-level explanations from brain processes to mental processes and behaviors, we have to be able to identify the lower level components (computational mechanisms) that are of constitutive relevance for the occurrence of higher level events (mental occurrences, behaviors) in providing explanations of how a manipulation of one level may impact the other [10]. Take the example of depression, for instance: an adequate computational description of the way that neural mechanisms implement mental operations might allow us to understand how antidepressant drugs, with their effects on the former, would thereby influence the latter. The same would be true in reverse, if we were, for instance, to measure the neural consequences of a psychological intervention. The consilience made by discovering an implementation that helps us develop an inter-level understanding results from an explanatory relationship that is constitutive rather than causal, as it points towards the constitutively relevant components of higher and lower-level entities and their constitutive dependency relationships. Thus, to understand how we can explain how a seemingly normal psychological and/or neural system can possess an underlying trait that makes it fail catastrophically and instantiate psychopathologically relevant states in the face of particularly maladaptive personally- or environmentally mediated triggering events requires us to embrace a constitutive attitude towards the implementational level descriptions of the system. Doing so considerably enriches the self-understanding of our scientific practice in computational psychiatry. This distinction provides us with an additional, theoretically meaningful, and more fine-grained conceptual framework to identify and reflect upon the nature of explanations in computational psychiatry that are nested into what Bennett et al. combined together into the category of explanatory modeling.

By providing this additional theoretical differentiation to describe existing modeling practices within computational psychiatry, we go beyond the already useful terminology proposed by Bennett et al. It should, however, be noted that our intention, as it was for Bennett et al., is descriptive rather than prescriptive. We do not argue that scientists should give up causal modeling for constitutive modeling. Rather, both types of modeling have their place and use, and indeed the latter cannot be constructed without at least some insight into the former.

## Benefits of constitutive models

Models that provide causal explanation are far more widespread in science as a whole than models providing constitutive explanations. Why should making this distinction, especially for computational psychiatry, be relevant?

We suggest that constitutive models have both epistemic and pragmatic benefits. As discussed above, the most general epistemic benefit of constitutive models is that they provide us with inter-level explanations, telling us what lower-level computational process a higher-level phenomenon constitutively depends on. However, this has further epistemic benefits, including their contribution to system diagnostics. If we have a way to identify dispositions in a system in terms of its components and constellation, we do not have to make the system produce the exact dysfunction we want to diagnose to compare our model against it, which would normally be the case if we wanted to assess a causal model of the system. Take the case of extreme risk aversion, which can be considered to be an aspect of many different psychiatric disorders including depression and generalized anxiety. We might be able to assess the nature of this disposition by the modest experiment described earlier (betting on coin flips), rather than requiring a manipulation that is more naturalistically valid. This would then afford us with substantial experimental control, and avoid the danger of reactivating the psychiatric state. The hope is that by simulating weakened triggering conditions, diagnostic insights could be generated, without causing e.g. a full blown panic attack. Such approaches are indeed already being discussed and developed [9]. To clarify, the procedures by which the presence of the dispositions which provide the constitutive explanation of higher-level psychopathological features are investigated in such scenarios are in themselves capitalizing on the causal profile of the dispositions. The difference is that we do not need to trigger the full causal response but can come up with proxy scenarios that give us enough information about the presence of the relevant features to infer a disposition to realize a certain behavior in a relevant naturalistic scenario that would be psychopathological relevant.

A pragmatic benefit of constitutive models is that they can be simpler while still explaining the target phenomenon. Telling a causal story about how an input produces a system’s output at a given level and grain of description will necessarily contain all parts and relationships also mentioned in the constitutive explanation, as these become parts of the exercised causal chain if the disposition is exercised. However, beyond this, a causal story will usually have to provide additional information about what is happening in the system after the input before the disposition’s activation and after, until the output is produced [11]. In most cases this will make a causal model more complex. So where simplicity is required, because it is not attainable to grasp the whole causal story, constitutive modeling provides a viable alternative.

Moreover, we should consider an additional, clinically relevant, feature. It has been reported that biological explanations, couched, for instance, in neural terms, decrease the empathy of clinicians with their patients [12]. This is ethically problematic, but also risks harming a good working relationship between patient and therapist, which in turn is a relevant predictor of better clinical outcomes [13]. Constitutive explanations might be helpful to mitigate any such loss of empathy, as they will often point out specific dispositional features which can be understood more naturally (‘risk aversion’) than can causal explanations, in their fuller, distancing, detail.

However, constitutive explanations are not without challenges. As Ylikoski [14] points out, providing constitutive explanations requires us to make some tough decisions. We have to determine the background conditions – the constitutive field – under which we consider the dispositional explanation to apply, as a disposition of a system being triggered may depend on its circumstantial context. Moreover, it may be a hard task to determine which exact component of a system should be taken into account for a constitutive model. Not every component of a system should be expected to have explanatory relevance for its dispositions, so methods to identify these components must be worked out in the background. With the problem of identifying the explanatory relevant components comes the task of deciding on the level of granularity or detail they should be described. In the end, an answer might be chosen based on pragmatic factors – for example, the level of description at which we are able to find responsible parts at all given our methods or the levels that are more easily accessible for cases in which we wish to assess an explanation to hold. Other concerns may have a more restricted scope, such as the observation that constitutive explanations, by not providing us with a step by step causal story, fail to encompass certain factors, such as typically the temporal order of processes in a system that determine its output.

## Conclusion

In this piece we argued that the “Two Cultures of Computational Psychiatry” are not the whole story. One of its cultures, explanatory modeling, can and should be considered to consist of two subcultures: causal modeling and constitutive modeling. Though causal modeling is perhaps the more widely practiced kind of explanatory modeling in computational psychiatry, constitutive modeling has its place due to its epistemic and pragmatic benefits. Distinguishing them is important for the methodological self-understanding of the discipline of computational psychiatry, interesting from a perspective of philosophy of science to make intelligible what explanatory strategies are employed in the field, and potentially relevant for guiding future research.
